# Frequent Periodic Leg Movement During Sleep Is an Unrecognized Risk Factor for Progression of Atrial Fibrillation

**DOI:** 10.1371/journal.pone.0078359

**Published:** 2013-10-16

**Authors:** Mahek Mirza, Win-Kuang Shen, Aamir Sofi, Canh Tran, Ahad Jahangir, Sulaiman Sultan, Uzma Khan, Maria Viqar, Chi Cho, Arshad Jahangir

**Affiliations:** 1 Center for Integrative Research on Cardiovascular Aging (CIRCA), Aurora University of Wisconsin Medical Group, Milwaukee, Wisconsin, United States of America; 2 Division of Cardiovascular Diseases, Mayo Clinic, Scottsdale, Arizona, United States of America; 3 Division of Cardiovascular Diseases, Mayo Clinic, Rochester, Minnesota, United States of America; Scuola Superiore Sant'Anna, Italy

## Abstract

Sleep apnea has been recognized as a factor predisposing to atrial fibrillation recurrence and progression. The effect of other sleep-disturbing conditions on atrial fibrillation progression is not known. We sought to determine whether frequent periodic leg movement during sleep is a risk factor for progression of atrial fibrillation. In this retrospective study, patients with atrial fibrillation and a clinical suspicion of restless legs syndrome who were referred for polysomnography were divided into two groups based on severity of periodic leg movement during sleep: frequent (periodic movement index >35/h) and infrequent (≤35/h). Progression of atrial fibrillation to persistent or permanent forms between the two groups was compared using Wilcoxon rank-sum test, chi-square tests and logistic regression analysis. Of 373 patients with atrial fibrillation (77% paroxysmal, 23% persistent), 108 (29%) progressed to persistent or permanent atrial fibrillation during follow-up (median, 33 months; interquartile range, 16-50). Compared to patients with infrequent periodic leg movement during sleep (n=168), patients with frequent periodic leg movement during sleep (n=205) had a higher rate of atrial fibrillation progression (23% vs. 34%; p=0.01). Patients with frequent periodic leg movement during sleep were older and predominantly male; however, there were no significant differences at baseline in clinical factors that promote atrial fibrillation progression between both groups. On multivariate analysis, independent predictors of atrial fibrillation progression were persistent atrial fibrillation at baseline, female gender, hypertension and frequent periodic leg movement during sleep. In patients with frequent periodic leg movement during sleep, dopaminergic therapy for control of leg movements in patients with restless legs syndrome reduced risk of atrial fibrillation progression. Frequent leg movement during sleep in patients with restless legs syndrome is associated with progression of atrial fibrillation to persistent and permanent forms.

## Introduction

Atrial fibrillation (AF), a common arrhythmia associated with increased morbidity and mortality, is increasingly recognized to be affected by sleep disturbances, particularly obstructive sleep apnea [1-3]. Restless legs syndrome (RLS) [4], another common cause of disturbed sleep, affects more than 12 million people in the United States [5]. However, no information is available on the effect of sleep-related movement disorders on AF. Periodic leg movement during sleep (PLMS) is common in patients with RLS and can be quantified by overnight polysomnography [6,7]. These movements manifest as involuntary leg-jerking movement during sleep, leading to disturbed sleep without the hypoxemia or changes in intrathoracic pressure seen with obstructive sleep apnea [8-12]. However, repeated arousals from sleep are associated with an increased sympathetic drive and lead to mental and physical stress with nocturnal fluctuations in blood pressure, heart rate and other hemodynamic effects [12-14] that can adversely affect cardiac electrophysiology [15,16] and promote structural remodeling [13,14,17]. Therefore, we hypothesized that patients with RLS and frequent PLMS may be at higher risk of progression of AF compared to those with infrequent PLMS. The objective of this study was to assess the impact of the severity of PLMS, and examine the association of periodic movement index on the progression of AF and whether treatment of RLS influences progression of AF in this patient population.

## Material and Methods

### Patient population

For this retrospective study, consecutive patients with a clinical suspicion of RLS [6,18] referred for polysomnography from January 2000 to August 2007 were identified from their medical records (International Classification of Diseases-9 code 333.94). Those with a history of paroxysmal or persistent AF and long-term follow-up were included in the study. Atrial fibrillation was considered paroxysmal if episodes were self-terminating, persistent if AF lasted for more than 7 days or required electrical or chemical cardioversion for termination or an antiarrhythmic agent for prevention of recurrence and permanent if electrical cardioversion failed, was not attempted or the patient remained in AF for more than a year [1,3,19]. The type of AF was determined by a thorough review of patient records defining symptoms and documenting rhythm before the sleep study and during follow-up. Patients with end-stage renal disease, severe neuropathy, Parkinson’s disease or severe anemia and those with Class III or IV heart failure were excluded. Participants did not provide their written or verbal informed consent to participate in this study as it was performed in a retrospective manner using data stored in medical records. No study interventions were performed and all analyses were performed using de-identified data, thus, no informed consent was required. The Mayo Clinic Institutional Review Board and the ethics committee approved the study and its consent procedure.

### Polysomnography

Overnight sleep study was conducted at the Mayo Clinic sleep laboratory using a computerized polysomnography device with simultaneous recordings of electroencephalogram, electrooculogram, electrocardiogram, submental and bilateral leg electromyogram, nasal and oral airflow, oxygen saturation (pulse oximetry), snoring, chest and abdominal respiratory movement and body position. Polysomnography results were scored according to standard criteria [20], and the results were interpreted by physicians with expertise in sleep medicine.

Periodic leg movement during sleep was defined on polysomnography according to the American Sleep Disorders Association criteria [21] as bursts of muscle activity in the anterior tibialis electromyogram of 0.5- to 5.0-seconds duration, >25% of the amplitude of the calibration level and as part of a series of ≥4 movements separated by 5 to 90 seconds. Periodic leg movement during sleep was scored only if unrelated to respiratory events during sleep. The frequency of PLMS was quantified by a periodic movement index, calculated as the number of movements per hour of total sleep time, with ≥5 movements per hour considered abnormal [22]. Periodic leg movement during sleep was classified as frequent if the periodic movement index was >35 movements/h or infrequent if ≤35 movements/h as defined using the receiver operating characterictic curve in our previous study [17], with the highest sensitivity and 1-specificity values of periodic movement index >35/h correlating with adverse cardiac structural changes and poor cardiovascular outcomes (area under the receiver operating characteristic curve 0.703; 95% confidence interval 0.664-0.740, p<0.001). Movements followed by an arousal from sleep were considered relevant in the generation of sleep disturbances and were additionally classified using the movement-related arousal index, calculated as the number of PLMS-related arousals per hour of total sleep time. Obstructive sleep apnea was defined by a reduction in airflow to <20% of baseline lasting for ≥10 seconds, and associated with respiratory efforts, and hypopnea as a reduction in airflow by 50% for ≥10 seconds accompanied by ≥4% oxygen desaturation or arousal, or both [10]. The apnea-hypopnea index was defined as the sum of hypopneas and apneas per hour of sleep time, and used as the summary statistic for sleep-disordered breathing.

### Follow-up

Follow-up information was obtained from a comprehensive medical record system at Mayo Clinic of all patients’ clinic visits or hospitalization and communication with their primary referring physician. Electrocardiograms were routinely obtained during follow-up visits or when indicated by symptoms suggestive of arrhythmia recurrence; 24-hour Holter monitoring and echocardiogram also were obtained when required. Atrial fibrillation progression at follow-up was defined as arrhythmia persistence with worsening of symptoms requiring evaluation or need for additional antiarrhythmic therapy to maintain sinus rhythm (paroxysmal to persistent AF), failed attempts at electrical cardioversion to restore sinus rhythm or when a rhythm control approach was not further pursued (paroxysmal or persistent to permanent AF) [1,3,19].

### Statistical analysis

Baseline clinical, echocardiographic and polysomnographic parameters were compared between the two groups to correlate the severity of PLMS to the progression of AF. Continuous variables were summarized as mean ± standard deviation; discrete variables were described as frequency and percentage. Wilcoxon rank-sum test was used to test for differences in continuous variable between two groups and chi-square tests were used to test for differences between two categorical variables. Since our outcome of interest, AF progression (Yes/No), is binary, a multivariate logistic regression model was used to examine the factors that are associated with arrhythmia progression. Age, gender, AF type (paroxysmal/persistent), history of hypertension, prior myocardial infarction or history of heart failure, periodic movement index (≤35/hr or >35/hr), apnea-hypopnea index (≤15 or >15) and movement-related arousal index were the covariates considered for analysis. A stepwise selection procedure with an entry and removal p-value criteria of 0.05 was used to select variables into the model. All statistical analyses were performed with SAS version 9.2 (SAS Institute Inc, Cary, North Carolina).

## Results

### Patient demographics

From January 2000 to August 2007, 4,951 consecutive patients with a clinical suspicion of RLS were referred for overnight polysomnography for sleep disturbance with an overall 8% prevalence of AF. Follow-up data was available in 382 patients with a diagnosis of AF, of which nine patients with permanent AF (5 in frequent and 4 in infrequent PLMS group) were excluded from the analysis. The remaining 373 patients with nonpermanent AF (77% paroxysmal and 23% persistent) were followed for the progression of AF. Patients were divided into those with frequent (periodic movement index >35/h) and infrequent PLMS (periodic movement index ≤35/h). Table 1 summarizes baseline characteristics of the overall study population and of the frequent and infrequent PLMS groups. No significant differences were found at baseline between the groups in the presence of major comorbidities that could potentially increase the risk for AF progression, such as hypertension, diabetes mellitus, coronary artery disease, myocardial infarction, heart failure, left ventricular ejection fraction or use of Class I or III antiarrhythmic agents. Both groups had increased left atrial volume index, which was not significantly different between the two groups (p=0.15). A trend toward a higher proportion of patients with persistent AF seen in the frequent PLMS group did not reach statistical significance (p=0.08). Patients with frequent PLMS were older (p=0.01) and predominantly male (p=0.001) with a higher proportion treated with beta blockers (p=0.02) and dopaminergic therapy for a movement disorder (p=0.01) than those with infrequent PLMS.

**Table 1:** Baseline clinical and echocardiographic characteristics of study population.

Characteristic*	**Overall (n=373)**	**Periodic Movement Index ≤35/hr (n=168)**	**Periodic Movement Index >35/hr (n=205)**	**p-Value**
Age, yrs	69 ± 11	67 ± 12	70 ± 10	0.01
Female	130 (35)	78 (46)	52 (25)	<0.001
Body mass index	32 ± 8	33 ± 8	32 ± 7	0.06
Paroxysmal atrial fibrillation	288 (77)	137 (81)	151 (74)	
Persistent atrial fibrillation	85 (23)	31 (19)	54 (26)	0.08
Hypertension	291 (78)	131 (78)	160 (78)	0.98
Diabetes mellitus	182 (49)	85 (51)	97 (47)	0.52
Hyperlipidemia	260 (70)	116 (69)	144 (70)	0.80
Coronary artery disease	132 (35)	51 (30)	81 (39)	0.06
Myocardial infarction	78 (21)	34 (20)	44 (21)	0.77
Congestive heart failure	34 (9)	16 (9)	18 (9)	0.80
Stroke	34 (9)	14 (8)	20 (10)	0.63
COPD	22 (6)	11 (6)	11 (5)	0.50
Chronic renal insufficiency	43 (11.5)	18 (11)	25 (12)	0.85
RLS medication	153 (41)	55 (33)	98 (48)	0.01
Dopaminergic agonists	124 (33)	44 (26)	80 (39)	
Non-dopaminergic agonists	29 (8)	11 (7)	18 (9)	
Antiarrhythmics	75 (20)	35 (21)	40 (19)	0.70
β-blockers	197 (53)	75 (45)	122 (59)	0.02
Calcium channel blockers	63 (17)	30 (18)	33 (16)	0.68
**Echocardiographic characteristics†**				
LV ejection fraction, %	54 ± 14	55 ± 14	54 ± 14	0.61
Left atrial volume index	49 ± 21	47 ± 23	51 ± 21	0.15
LV end-diastolic size	52 ± 9	51 ± 9	52 ± 9	0.12
LV end-systolic size	36 ± 11	35 ± 11	37 ± 11	0.15

* Categorical values are presented as number (percentage); continuous variables as mean ± standard deviation.

† Echocardiographic data was available in 233 patients.

COPD = chronic obstructive pulmonary disease; LV = left ventricular; RLS = restless legs syndrome.

### Polysomnographic variables

Results of the overnight polysomnographic parameters in the two groups, including sleep architecture, arousal patterns due to leg movements and breathing disorder, are summarized in Table 2. The mean periodic movement index in the frequent PLMS group was 94 ± 42 compared to 11 ± 11 in the infrequent group (p<0.001). The overall total sleep time and proportion of patients with the time spent in the early Stages 1/2 non-rapid eye movement or rapid eye movement sleep were not significantly different between the two groups. However, the number of patients in the frequent PLMS group who spent time in the deep (slow-wave) Stage 3/4 non-rapid eye movement sleep was significantly smaller than in the infrequent PLMS group (p=0.02). The pattern of arousals from sleep between the two groups also was quite different, with the frequent PLMS group predominantly demonstrating arousals due to leg movement (34 ± 24 vs. 7 ± 11; p<0.001) compared with the increase in breathing-related arousals seen more in the infrequent PLMS group (61 ± 30 vs. 51 ± 27; p<0.01). The apnea-hypopnea index (p=0.55) or the prevalence of obstructive sleep apnea defined as an apnea-hypopnea index ≥15/h (p=0.32) were not significantly different between the two groups.

**Table 2:** Polysomnographic variables of study population.

Characteristic*	**Overall (n=373)**	**Periodic Movement Index ≤35/hr (n=168)**	**Periodic Movement Index >35/hr (n=205)**	**p-Value**
TST, min	177 ± 82	181 ± 92	173 ± 73	0.90
TST in Stage 1	33 ± 25	34 ± 27	32 ± 22	0.76
TST in Stage 2	97 ± 55	95 ± 57	100 ± 53	0.3
TST in Stage 3/4	26 ± 30	31 ± 35	22 ± 26	0.02
REM sleep	20 ± 25	22 ± 27	19 ± 22	0.78
Periodic movement index	57 ± 54	11 ± 11	94 ± 42	<0.001
Movement-related arousal index	22 ± 23	7 ± 11	34 ± 24	<0.001
Breathing-related arousal index	55 ± 29	61 ± 30	51 ± 27	<0.01
AHI, frequency/hr	22 ± 24	25 ± 26	21 ± 22	0.55
AHI ≥15/hr	176 (47)	74 (44)	92 (46)	0.32

* Categorical values are presented as number (percentage); continuous variables as mean ± standard deviation.

AHI = apnea-hypopnea index; REM = rapid eye movement; TST = total sleep time.

### Atrial fibrillation progression and predictors of progression

During a median 33 months (interquartile range, 16-50 months) follow-up, AF became more refractory in 108 patients (29%). Of the 288 patients with paroxysmal AF, 221 (77%) remained in paroxysmal AF as of the last follow-up, whereas 63 (22%) progressed to persistent AF and 4 (1%) to permanent AF. Of the 85 patients with persistent AF at baseline, 41 (48%) progressed to a more resistant form either requiring additional antiarrhythmic agents for symptomatic recurrences or to permanent AF. There was a significantly higher rate of AF progression seen in the frequent PLMS group than the infrequent group (34% vs. 23%; p=0.01). The univariate and multivariate predictors of AF progression with odds ratio (OR), 95% confidence interval (CI) and p-values are summarized in Table 3. Older age, female gender, persistent AF, history of hypertension, frequent PLMS (periodic movement index >35/h) and movement-related arousal index were univariate predictors. After correction for baseline differences on multivariate analysis, periodic movement index >35/h (OR, 2.24; 95% CI, 1.33-3.78; p=0.003), presence of persistent AF (OR, 3.51; 95% CI, 2.04-6.05; p<0.0001), female gender (OR, 2.26; 95% CI, 1.34-3.81; p=0.002) and history of hypertension (OR, 2.25; 95% CI, 1.16-4.36; p=0.01) were independent predictors of AF progression.

**Table 3:** Univariate and multivariate predictors of atrial fibrillation progression.

**Characteristic**	**OR (95% CI)**	**p Value**
**Univariate Predictors**		
Persistent atrial fibrillation	3.22 (1.92-5.39)	<0.0001
Periodic movement index >35/hr	1.88 (1.17-3.02)	0.009
Age, yrs	1.03 (1.01-1.05)	0.01
Hypertension	1.98 (1.07-3.66)	0.02
Female	1.7 (1.07-2.72)	0.03
Movement-related arousal index	1.01 (1-1.02)	0.04
Myocardial infarction	0.64 (0.35-1.16)	0.14
Coronary artery disease	0.73 (0.45-1.18)	0.20
Stroke	0.74 (0.32-1.68)	0.47
Apnea-hypopnea index ≥ 15	1.08 (0.69-1.7)	0.74
Congestive heart failure	0.87 (0.39-1.94)	0.74
**Multivariate Predictors**		
Persistent atrial fibrillation	3.51 (2.04-6.05)	<0.0001
Periodic movement index >35/hr	2.24 (1.33-3.78)	0.003
Female	2.26 (1.34-3.81)	0.002
Hypertension	2.25 (1.16-4.36)	0.01

CI = confidence interval; OR = odds ratio.

### Effect of restless leg syndrome treatment on atrial fibrillation progression

The effect of RLS treatment with dopaminergic drugs that reduce abnormal leg movements during sleep on the rate of AF progression was determined in 153 patients in whom such treatment was prescribed. The number of patients on treatment for RLS symptoms was significantly higher in the frequent PLMS group (p=0.01; Table 1). Ninety eight patients (48%) with frequent PLMS received treatment with dopaminergic agonists (39%: Levodopa plus Carbidopa) or non-dopaminergic agonists (9%: Ropinirole or Pramipexole), whereas 55 patients (33%) with infrequent PLMS received treatment with dopaminergic agonists (26%: Levodopa plus Carbidopa) or non-dopaminergic agonists (7%: Ropinirole or Pramipexole) for the relief of RLS symptoms. There were no differences in the progression of AF between the treated versus untreated in the infrequent PLMS group for which dopaminergic drugs were given for symptom control (p=0.33). However, in the frequent PLMS group, the overall rate of AF progression was 2.7-fold lower (11.6%) in the group treated with dopaminergic drugs versus those who did not receive treatment (32%; p=0.01). In a subgroup analysis of patients on treatment with dopaminergic therapy, treatment for PLMS was a predictor for reduction in the risk of AF progression in patients with frequent PLMS (OR, 0.18; 95% CI, 0.06-0.55; p=0.003).

## Discussion

The main finding of this study is that frequent periodic leg movements during sleep in patients with restless legs syndrome are associated with a greater progression of AF than in those with infrequent PLMS. The association between frequent PLMS and the risk for progression of AF persisted even after correction for factors known to modulate AF progression, such as advanced age, hypertension, coronary artery disease, heart failure or obstructive sleep apnea. This association is further strengthened by the observation that use of medications that reduce leg movements during sleep decrease the rate of progression in those with frequent PLMS. These findings are of potentially high clinical significance as recognition of another modifiable risk factor for AF progression – i.e. frequent PLMS, especially in RLS patients – could help reduce the overall burden of AF in the elderly, a population with high prevalence of both RLS [4,6] and AF [3,19,23-25].

Sleep plays an important role in maintaining normal body homeostasis, and more evidence is accumulating regarding the adverse impact of sleep disturbance on cardiovascular hemodynamics [26] as well as structural and functional remodeling [17]. Sleep-disordered breathing, particularly obstructive sleep apnea, has been well-recognized as a factor that contributes to incident and recurrent AF [27,28], but the effect of other conditions that result in fragmented sleep on AF have not been studied [29,30]. To our knowledge, the relationship between RLS and AF progression has not been described previously, and this is the first report recognizing such an association. Restless leg syndrome is a clinical diagnosis that requires fulfillment of four essential criteria as defined by the International Restless Legs Syndrome Study Group [6]: a desire to move the limbs, often associated with paresthesias or dysesthesias; symptoms that are worse or present only during rest and are temporarily relieved by activity; motor restlessness; and worsening of symptoms at rest or nocturnally. Since these strict clinical criteria cannot be assessed from a retrospective review of medical records and are more subjective in nature, we relied on objective documentation of the severity of PLMS on polysomnography as defined by the American Sleep Disorders Association criteria [21], which is a particular strength of our study. Periodic leg movement during sleep is present in about 80% of patients with RLS [31], with frequent PLMS causing repeated awakenings and sleep disruption reported as the most frequent reason for patients to seek medical assistance [5,14,32]. We recently described the association of frequent PLMS with structural remodeling of the heart promoting left ventricular hypertrophy and adverse cardiovascular outcomes, including a higher incidence of heart failure and mortality in those with frequent PLMS [17]. After adjustment for known risk factors, periodic movement index >35/h was an independent predictor for adverse cardiovascular outcomes. We now extend these findings to patients with RLS and paroxysmal or persistent AF in whom the presence of frequent PLMS on polysomnography was associated with higher rates of AF progression compared to those with infrequent PLMS. These findings are of high clinical relevance because RLS is a common condition that affects more than 12 million individuals in the United States, with an estimated populace prevalence of 5-18% that increases with advancing age, thus predominantly affecting a population that is also at high risk for AF and its complications [3-6,24].

A heterogeneous condition with variable etiology, natural history and prognosis [19,33], AF progresses with time in most patients, where infrequent self-terminating episodes that respond to rhythm controlling medication become more frequently sustained, with symptoms refractory to treatment, ultimately progressing to the permanent form [34-36]. In our study, patients with frequent PLMS were more likely to progress to require additional antiarrhythmic therapy for symptomatic recurrences or to permanent AF than those with infrequent PLMS. Periodic movement index >35/h remained an independent predictor even after correction for other known factors promoting AF progression. Persistent AF, female gender and hypertension were other factors independently predictive of AF progression in this population. Treatment for RLS with dopaminergic drugs was protective against AF progression in the frequent PLMS group. These observations are suggestive of a relationship between frequent PLMS and AF progression.

However, the precise mechanisms underlying increased progression of AF in patients with RLS are not known. A circadian pattern of symptoms that peak nocturnally is common for most patients with RLS [4] and is proposed to result in an altered neurohumoral milieu with heightened sympathetic tone [14] that correlates with the nocturnal timing of AF, particularly in patients without structural heart disease [1,4,9,37-39]. Oscillations in sympathetic activity and changes in vagal tone in patients with frequent PLMS [12,40] are important determinants of blood pressure and chronotropic responses during sleep [13,15,16,41] causing cardiac acceleration [42-44], and correlate with a higher magnitude of sympathetic activity assessed by heart rate variability and cortical arousals [15,45] with short awakenings documented by abrupt changes in electroencephalographic pattern [21]. Evidence of a link between electroencephalographic changes and cardiac activation during sleep have been provided by different studies [42-44,46]. This also may predispose to adverse cardiovascular remodeling that can lead to mechanical [17] and electrical alterations, increasing the risk of cardiovascular events [1,13,14,19,29,47,48] and AF progression. In patients with frequent PLMS, our data illustrates a reduced time of slow-wave Stage 3/4 sleep, considered to be the most “restorative” phase of sleep associated with the largest decline in sympathetic activity. A reduction in the time in this slow-wave phase of sleep was recently reported to predict increased incident hypertension [26] and could potentially increase predisposition to arrhythmogenesis in those with frequent PLMS. If frequent PLMS contributes to the progression of AF, then suppressing these movements with dopaminergic therapy could impede AF progression. This is consistent with our observations that the rate of AF progression was lower in those with frequent PLMS on dopaminergic therapy, which could be the result of a reduction in leg movements and improvement in the quality of sleep that in turn prevented nocturnal oscillations in blood pressure, hemodynamics and sympathetic activity, and resultant effect on cardiac acceleration, as was recently demonstrated for Pramipexole, a dopaminergic agent used for RLS [49].

Our observational study should be interpreted with limitations imposed by the retrospective study design. The results suggest an association of RLS to AF progression that needs to be confirmed in a large prospective study to minimize the effect of uncontrollable confounding factors that cannot be fully accounted for in a retrospective review. There were no significant differences between the frequent and infrequent groups in terms of hypertension, heart failure, coronary artery disease, prior myocardial infarction and left ventricular ejection fraction, factors known to promote AF progression. Assessment of a causal relationship between frequent PLMS and AF progression is outside the scope of this study. The purpose of the present study was to identify an association between the frequent leg movements during sleep and AF progression independent of known factors promoting this outcome. We relied on objective data of PLMS severity and arousals during sleep determined by polysomnography, which is a strength of this study compared to previous assessment of symptom severity from self-administered questionnaires [14]. Assessment of progression of AF in those who were treated with a dopaminergic agent provides additional evidence strengthening the association between frequent PLMS and progression of AF. The information about AF progression was determined from a close review of patients’ records documenting need for additional therapy for symptoms related to AF. Follow-up electrocardiogram was obtained based on symptoms or during routine follow-up examination, making it difficult to quantify the exact burden and duration of AF that may have underestimated the progression in asymptomatic patients. Since our study comprised a population of patients with a clinical suspicion of RLS who were referred for overnight polysomnography, it would be difficult to determine the true incidence of AF from a retrospective review of medical records, especially when electrocardiographic evidence was not routinely obtained in non-AF patients. A prospective cohort will be able to best address that question, providing a true estimate of the incidence of arrhythmia in this population.

## Conclusions

We report that frequent periodic leg movement during sleep in patients with clinical suspicion of restless leg syndrome is associated with the progression of atrial fibrillation. Since atrial fibrillation is the most common arrhythmia encountered in clinical practice, particularly in the elderly who also may have restless legs syndrome, another commonly prevalent condition [50], recognition of this association and underlying mechanisms predisposing to atrial fibrillation and its progression becomes important. With the rapid change in population demographics [51] and a projected increase in the prevalence of atrial fibrillation [2,24] and restless legs syndrome [5,6], it is important that mechanisms underlying atrial fibrillation development and progression in the elderly need to be better defined so preventive strategies can be implemented to reduce morbidity, death and cost associated with these conditions.
